# A cross-sectional study of university students’ mental health and lifestyle practices amidst the COVID-19 pandemic

**DOI:** 10.1371/journal.pone.0302265

**Published:** 2024-04-16

**Authors:** Reem Hoteit, Imad Bou-Hamad, Sahar Hijazi, Dinah Ayna, Maya Romani, Christo El Morr

**Affiliations:** 1 Clinical Research Institute, Faculty of Medicine, American University of Beirut, Beirut, Lebanon; 2 Department of Business Information and Decision Systems, Suliman S. Olayan School of Business, American University of Beirut, Beirut, Lebanon; 3 Faculty of Social Sciences, Lebanese University, Saida, Lebanon; 4 Department of Psychiatry, Faculty of Medicine, American University of Beirut Medical Center, Beirut, Lebanon; 5 Department of Family Medicine, Faculty of Medicine, American University of Beirut Medical Center, Beirut, Lebanon; 6 School of Health Policy and Management, York University, Toronto, Canada; Tehran University of Medical Sciences, ISLAMIC REPUBLIC OF IRAN

## Abstract

**Objectives:**

University students are regarded as the backbone of society, and their mental health during a pandemic may have a substantial impact on their performance and life outcomes. The purpose of this study was to assess university students’ mental health, specifically depression, anxiety, and stress, during Lebanon’s extended COVID-19 pandemic, as well as the sociodemographic factors and lifestyle practices associated with it.

**Methods:**

An online anonymous survey assessed the rates of mental health problems during COVID-19, controlling for socio-demographics and other lifestyle practices, in 329 undergraduate and graduate university students. Instruments utilized were the Patient Health Questionnaire (PHQ-9) for depression, the Beck Anxiety Inventory (21-BAI) for anxiety, and the Perceived Stress Scale (PSS-10) for stress. The study employed descriptive statistics and multiple logistic regression models to analyze the association between depression, anxiety, and stress with sociodemographic and lifestyle factors. Results were evaluated using adjusted odds ratios and confidence intervals, with a significance level of 0.05.

**Results:**

Moderate to severe rates of depression, anxiety and stress among students were reported by 75.9%, 72.2%, and 89.3%, respectively. The odds of anxiety and stress were higher among women compared to men. Students who used private counseling services had higher odds of anxiety and stress than those who did not. Overall rated health was a major predictor of depression and anxiety, with the "poor" and "fair" overall-reported health groups having higher odds than the "Excellent" group. When compared to those who did not smoke, students who increased their smoking intake had higher odds of depression, anxiety and stress. Students who reduced their alcohol consumption had lower odds of anxiety compared to those who did not consume alcohol. Students who reduced their physical activity had higher odds than those who increased it. Finally, students who slept fewer than seven hours daily had higher odds of depression than those who slept seven to nine hours.

**Conclusion:**

Our findings indicate a national student mental health crisis, with exceptionally high rates of moderate to severe depression, anxiety, and stress. Factors such as gender, university program, overall rated health, importance of religion in daily decisions, private counseling, smoking cigarettes, alcohol consumption, physical activity, and sleeping, were all found to have an impact on mental health outcomes. Our study highlights the need for university administrators and mental health professionals to consider targeted mental health programming for students, particularly for women and those with poor or fair overall perceived health.

## Introduction

Since the World Health Organization proclaimed a universal pandemic [1], the coronavirus disease outbreak (COVID-19) has spread globally with a substantial impact on people’s lives. COVID-19 had infected around 480 million individuals as of April 4, 2022, with 6 million deaths worldwide [2]. With the emergence of the Omicron variant, the number of infections are increasing exponentially, with around two million people infected per day in the first week of January 2022 [3]. Since the beginning of the pandemic, many governments have implemented a variety of anti-epidemic measures to contain the spread of the virus, such as restricting foreign nationals’ travel [4, 5], closing public areas, and shutting down entire transit systems [6]. Preventing the extremely contagious variant from spreading became the world’s top priority [7]. Nonetheless, the prolonged pandemic has raised concerns about the world population’s mental health [8], particularly in relation to the psychological effects of quarantine procedures that disrupted daily routines, such as the suspension of in-person activities, the adoption of distancing measures, and social isolation. The long duration of associated measures poses a host of challenges, obstructions, and risks to physical and mental health conditions including depression, anxiety and stress [9, 10]. While the entire population of the world is impacted, the subgroup of young adults, particularly college students, are thought to be particularly vulnerable.

Young people’s mental health has long been recognized as a global public health concern [11]. For example, student distress is both an individual and societal challenge, for loss in productivity at work and during their study is associated with major economic burdens [12]. Several studies have been conducted around the world to investigate the psychological effect of the COVID-19 pandemic on young people and students’ mental health [13–15]. Most studies discovered increased levels of anxiety, depression, and stress in various countries [16–19]. According to an online cross-sectional multicounty survey of Asian university students (Pakistan, China, India, Indonesia, Saudi Arabia, Malaysia and Bangladesh) conducted in 2021, 35.6% expressed mild to severe anxiety [20]. Additionally, Wang et al. 2020 found that 48.14% of undergraduate and graduate university students in the United States had moderate-to-severe depression, 38.48% had moderate-to-severe anxiety, and the majority of participants (71.26%) stated their stress levels had increased during the pandemic [21]. Research on university students during the COVID-19 pandemic from Bangladesh, Egypt, Ethiopia, Lebanon, Turkey, and Brazil reported substantial variation in the percentages of depression (21.2% in Ethiopia to 82.4% in Bangladesh), anxiety (27.7% in Ethiopia to 87.7% in Bangladesh), and stress (12.7% in Lebanon to 57.5% in Brazil) symptoms [22–26]. Furthermore, a systematic review and meta-analysis conducted by Wang et al. in 2020 assessing anxiety, depression, and stress prevalence among college students during the COVID-19 pandemic found that the prevalence of anxiety, depression, and stress was 29%, 37%, and 23%, respectively [16].

In Lebanon, the first confirmed COVID-19 case in the country was reported on February 21, 2020. In an attempt to flatten the curve, the government adopted multiple lockdowns between 2020 and 2021, giving authorities the legislative power to implement extraordinary measures against COVID-19, such as border closures (airport, sea, and land) and closures of public and private facilities [27, 28]. The population in Lebanon is around 7 million; since the beginning of the COVID-19 pandemic, the Lebanese Ministry of Public Health (MOPH) has confirmed around one million cases and more than 10 000 death as of April 4, 2022 [29]. To date, 49%, 43% and 24% of the Lebanese population over the age of 12 have received their first, second, and third doses of vaccine, respectively, making the immunization process sluggish [29]. This unstable epidemiological situation, particularly in light of the emergence of a new highly transmissible variant such as Omicron, has given rise to a slew of concerns, including an increase in infection fears and significant lifestyle changes as a result of lockdown measures, all of which have had an impact on the population’s psychological well-being and mental health [28]. The negative impact of the COVID-19 pandemic on mental health in Lebanon was demonstrated in tertiary referral hospital population [30], healthcare workers [31, 32], refugees [33], general population [7, 34], and young population (18 to 35 years) [35]. According to a study by Fawaz and Samaha in 2021, 17.9%, 13.8%, and 1.7% of students exhibited mild, moderate, and severe depressive symptoms, respectively; also, mild, moderate, severe, and extremely severe anxiety symptoms were found in 3.3%, 21.9%, 6.3%, and 2.3%, respectively; and 11% of students reported mild stress, while 1.7% reported moderate stress. However, Fawaz and Samaha’s study was conducted in April 2020, at the onset of the COVID-19 pandemic, when the impact of the pandemic on mental health was minimal; therefore, preliminary examination of depression, anxiety, and stress suggests that further inquiry on these issues is needed to better document, understand, and plan for appropriate mental health programming for students, especially in light of their increased vulnerability.

This study attempts to help fill the gap in the scarcity of literature on the impact of the pandemic on the mental health of students in Lebanon.

## Materials and methods

### Study design and participants

This cross-sectional survey was conducted in Lebanon using an online survey distributed to undergraduate and graduate university students. A link to the survey, with a study description, was sent to students and faculty via electronic platforms. Data was collected towards the end of the second year of the COVID-19 pandemic, particularly during the Omicron variant emergence (3 November 2021 and 7 February 2022). The sample of this study included 329 students, with the following inclusion criteria: undergraduate and graduate students who were 18 years of age or higher, enrolled at the American University of Beirut (a private university) or the Lebanese University (a public university) between Spring 2020–2021 and Fall 2021–2022.

The questionnaire was distributed in Arabic and English in Lebanon. Prior to filling out the survey, all participants provided written informed consent online. To adapt to the rapidly changing pandemic context and prioritize participant and researcher safety, we employed an online convenience sampling approach. This method was chosen over traditional in-person methods to minimize potential transmission, particularly given the rapid spread associated with the Omicron variant. The decision to use online distribution platforms aligns with previous methodologies adopted in COVID-19-related research [28, 36, 37]. No financial incentive was provided to the participants and anonymity was maintained to ensure the confidentiality and reliability of data. This study was conducted in full compliance with the provisions of the Declaration of Helsinki regarding research on human participants. Ethics approval for the study was obtained from the Institutional Review Board at the American University of Beirut (SBS-2021-0256) and the Research Ethics Board at York University in Canada (Certificate # e2021-327).

### Measures

#### Sociodemographic and other factors

Measured sociodemographic factors were age, gender, income, current program, nationality, relationship status, number of people living in the household. Lifestyle practices included cigarette and shisha smoking, alcohol intake, physical activity, sleeping patterns, internet usage, and overall health. Participants were also asked if they had sought private counseling or therapy from a clinical mental health professional, if they had tried mindfulness meditation, if they had followed COVID-19 preventive measures (wearing masks, handwashing, quarantining, etc.), if they had received a COVID-19 vaccine, and if they had kept up with COVID-19 updates. Finally, participants were asked if they had COVID-19 infection, if they believe that Corona virus and vaccination were the subject of a conspiracy, and if religion is important in their daily lives.

#### Mental health outcomes

*Depression (PHQ-9; Kroenke*, *2001)*. The Patient Health Questionnaire (PHQ-9) [38] is a brief 9-items, widely used, screening tool that is used to detect depression symptoms in community settings. The Diagnostic and Statistical Manual of Mental Disorders (DSM-IV), 4th Edition, was used to develop the PHQ-9. Prior to administration, each item is assessed for the prior two weeks: 0 = "not at all," 1 = "several days," 2 = "more than half the days," and 3 = "nearly every day," with a total score ranging from 0 to 27, and higher values indicating more severe depression. Minimum depression is indicated by a score of 0–4; mild depression 5–9; moderate depression 10–14; moderately severe depression 15–19; severe depression 20–27 [38]. "Feeling down, depressed, or hopeless," as well as "Poor appetite or overeating," are examples of scale items.

Participants with a score of 10 or above were assigned to the Possible Major Depressive Disorder (MDD) group, while those with a score of 9 or less were assigned to the Non-MDD group [38]. With a sensitivity of 80% and specificity of 92%, a total score of 10 or above indicated the possibility of serious depression [39, 40]. Additionally, PHQ-9 is a self-rating scale with strong reliability and validity for students [41, 42]. The Arabic-translated version of the PHQ-9, which has been validated, demonstrated good reliability with a Cronbach alpha of 0.88 [43]. In our study, the Cronbach’s alpha coefficient of the PHQ-9 was 0.901.

*Anxiety (Beck Anxiety Inventory (BAI); Beck et al*., *1988)*. Anxiety was assessed using the Beck Anxiety Inventory (BAI), a 21-item questionnaire that measures anxiety symptoms [44, 45]. Participants must rate themselves on a 0–3 scale, with zero indicating "Not at all" and three indicating "Severely-It bothered me a lot," with a maximum score of 63 and a minimum score of zero. Minimal anxiety is a score of 0–7, mild anxiety 8–15, moderate anxiety 16–25, and severe anxiety 26–63 [46]. A score of 16 is considered the clinical cut-off for anxiety [47]. The items reflect frequent anxiety symptoms, such as worry of the worse happening, increase in heart rate, fear of losing control, and fear of dying. The BAI demonstrated high internal consistency (Cronbach’s alpha = 0.94) and acceptable reliability throughout an average time lapse of 11 days (r = 0.67) in earlier research [48]. The Arabic-translated version of the 21-BAI scale has been validated among university students in Kuwait, with Cronbach’s alpha estimated to be between 0.83 and 0.90 [49]. In our study, the Cronbach’s alpha coefficient of the BAI scale was 0.944.

*Stress (Perceived Stress Scale (PSS); Cohen*, *Kamarck & Mermel-stein*, *1983)*. Generalized stress was measured using the 10-item Perceived Stress Scale (PSS) that measures symptoms of stress [50]. It has negative elements that test lack of control and unpleasant affective reactions, as well as positive elements that examine the ability to cope with current stressors. Item examples include, ‘How often have you felt nervous or stressed?’ and ‘How often have you felt confident about your ability to handle your personal problems?’ People rated how often they had experienced these feelings during the past month on a five-point Likert scale from 0 = never to 4 = very often. PSS-10 scores were obtained by reversing the scores on the four positive items; the items were 4, 5, 7 and 8. Total scores vary from 0 to 40, with 0–13 indicating mild stress, 14–26 indicating moderate stress, and 27–40 indicating high stress. In this study, high perceived stress associated with COVID-19 was defined as a score of 27 or above. This cut-off point has been used in a previous study [51].

The PSS is a simple global stress measure that has been proven to be reliable and valid in a variety of settings and languages [52–55]. In particular, the PSS-10 questionnaire was validated to assess stress among university students in a study conducted in China [56]. The Arabic version of the PSS-10 was validated in a study conducted in Lebanon, demonstrating good Cronbach’s alpha reliability (0.74) [57]. The Cronbach’s alpha coefficient of the PSS-10 scale was 0.846 in this study.

### Data analysis

Descriptive statistics were used to summarize the outcome variables, sociodemographic and other self-reported factors. Continuous variables were summarized as means and standard deviations (SDs), while categorical variables were summarized as frequencies and percentages. The study’s dependent variables: depression, anxiety, and stress were dichotomized based on the cut-off points. Three multiple logistic regression models were performed to model the dependent variables using the independent sociodemographic variables and lifestyle factors.

Using simple and multiple logistic regression, the unadjusted and adjusted odds ratios (U-OR; A-OR), as well as the 95% confidence interval (95% CI), were estimated. The Hosmer-Lemeshow test was used to evaluate the logistic models’ fit. For the analysis, the R programming language was used (version 4.1.2). The level of statistical significance was set at 0.05.

## Results

### Socio demographic characteristics

The current study included 329 students. Table 1 summarizes the descriptive statistics for the study participants’ characteristics. The mean (SD) age of the participants was 24.99 (7.39) years. The majority of participants were females (63.8%). Students were enrolled in a variety of university programs, with undergraduate students accounting for 43% of the sample. More than two-thirds (77.5%) of participants had a household monthly income of 450 dollars or less. Approximately 60% of students considered their overall health to be good, very good, or excellent. Sixty-four percent of the respondents stated that religion is important in their daily lives. Corona virus and vaccination were the subject of a conspiracy, according to 14% of participants. Furthermore, the majority of students (73.6%) followed COVID-19 prevention guidelines, and about a quarter of them were infected with COVID-19. Private counseling was received by more than half of the students (57.4%).

**Table 1:** Socio-demographic characteristics of university students and bivariate relationships (N = 329).

		Depression		Anxiety		Stress	
**N**		329		324		326	
**Mean (SD)**		10.18(6.83)		18.81(14.42)		21.97(7.30)	
	**n (%)**	U-OR	**P-value**	U-OR	**P-value**	U-OR	**P-value**
**Age (Mean (SD))**	24.99 (7.39)	0.94	**0.001**	0.97	**0.04**	0.97	0.139
**Gender**							
Men	77(23.4)	ref		ref		ref	
women	210(63.8)	1.35	0.268	2.05	**0.007**	2.20	**0.019**
*Missing*	42(12.8)						
**Relationship status**							
Not in a relationship	172(52.3)	1.51	0.088	1.15	0.553	1.35	0.267
In a relationship	118(35.9)	ref		ref		ref	
*Missing*	39(11.9)						
**University program**							
Undergraduate degree	143(43.5)	2.12	**0.001**	1.62	**0.044**	1.77	**0.031**
Certificate program	14(4.3)	4.55	**0.01**	3.93	**0.04**	2.55	0.103
Graduate program (MA or MSc)	141(42.9)	ref		ref		ref	
PhD Program	12(3.6)	0.91	0.88	0.21	0.05	2.17	0.982
MD program	19(5.8)	1.32	0.57	0.78	0.616	1.572	0.395
*Missing*							
**GPA status during the pandemic**							
No change	103(31.3)	0.78	0.373	0.78	0.375	0.71	0.281
Decreased	103(31.3)	1.36	0.248	1.07	0.782	0.81	0.471
Increased	123(37.4)	ref		ref		ref	
**Income (in USD)**							
≤450	255(77.5)	1.28	0.352	2.00	**0.010**	1.03	0.899
>450	74(22.5)	ref		ref		ref	
**Overall rated health**							
Poor	29(8.8)	74.99	**<0.001**	335.90	**<0.001**	2.97	0.979
Fair	100(30.4)	23.29	**0.003**	30.85	**0.001**	1.04	0.981
Good	121(36.8)	6.37	**0.07**	7.89	0.050	4.07	0.982
Very good	66(20.1)	3.52	0.24	7.80	0.055	2.47	0.982
Excellent	13(4.0)	ref		ref		ref	
**Importance of religion in daily decisions**							
Not important	70(21.3)	ref		ref			
Important	213(64.7)	0.67	0.160	1.10	0.709	0.61	0.095
*Missing*	46(14.0)						
**Conspiracy behind COVID virus/vaccine**							
Disapprove	117(35.6)	0.56	0.098	0.88	0.727	0.71	0.358
Neither approve nor disapprove	117(35.6)	0.69	0.293	0.95	0.882	0.81	0.566
Approve	49(14.9)	ref		ref			
*Missing*	46(14.0)						
**Adherence to COVID-19 preventive measures**							
No	41(12.5)	2.07	**0.0348**	1.62	0.165	0.80	0.587
Yes	242(73.6)	ref		ref			
*Missing*	46(14.0)						
**Infected with COVID-19**							
No	197(59.9)	1.01	0.958	1.29	0.314	0.92	0.774
Yes	86(26.1)	ref		ref		ref	
*Missing*	46(14.0)						
**Private Counseling**							
No	140 (42.6)	ref		ref		ref	
Yes	189 (57.4)	1.24	0.06	1.77	**0.010**	2.09	**0.004**

U-OR depicts the unadjusted bivariate odds ratio.

### Lifestyle practices

Regarding lifestyle practices during the pandemic (Table 2), around two-thirds (63.5%) of the participants followed a healthy diet. An increase in cigarette and shisha smoking, as well as alcohol usage, was self-reported by around 12% of the respondents. Physical activity decreased for nearly half of the students, while it increased for 31%. A third of the participants (32.2%) slept for fewer than seven hours, while 17.3% slept for more than nine hours. The majority of participants (70%) used the internet for at least 3 hours daily.

**Table 2:** Association between university students’ mental health outcomes and lifestyle practices during the pandemic and bivariate relationships.

		Depression		Anxiety		Stress	
	n (%)	U-OR	P-value	U-OR	P-value	U-OR	P-value
**Follow healthy diet**							
No	120(36.5)	2.18	**<0.001**	1.46	0.100	2.75	**<0.001**
Yes	209(63.5)	ref		ref		ref	
**Cigarette smoking**							
No practice	279(84.8)	ref		ref		ref	
Reduced	8(2.4)	0.81	0.786	3.02	0.180	1.69	0.47
Increased	42(12.8)	2.72	**0.004**	2.51	**0.010**	1.91	**0.05**
**Shisha smoking**							
No practice	262(79.6)	ref		ref		ref	
Reduced	27(8.2)	0.62	0.277	0.96	0.935	0.56	0.269
Increased	40(12.2)	1.88	0.066	1.21	0.569	1.20	0.616
**Alcohol consumption**							
No practice	252(76.6)	ref		ref		ref	
Reduced	38(11.6)	0.86	0.684	0.55	0.090	1.03	0.921
Increased	39(11.9)	1.02	0.952	1.36	0.370	1.00	0.997
**Physical activity**							
No practice	63(19.1)	2.50	**0.005**	1.38	0.311	2.68	**0.008**
Reduced	164(49.8)	1.90	**0.013**	1.23	0.405	2.45	**0.004**
Increased	102(31.0)	ref		ref		ref	
**Sleeping hours**							
**<7**	106(32.2)	2.46	**<0.001**	1.77	**0.023**	1.32	0.315
7–9	166(50.5)	ref		ref	0.726	ref	
>9	57(17.3)	2.90	**<0.001**	1.11		2.63	**0.002**
**Internet use (in hours)**							
<1	12(3.6)	ref		ref		ref	
[1–2[	31(9.4)	1.26	0.744	1.58	0.502	0.69	0.623
[2–3[	59(17.9)	0.95	0.939	0.68	0.553	0.360	0.151
[3–4[	57(17.3)	1.35	0.652	1.28	0.698	0.47	0.291
≥4	170(51.7)	2.30	0.186	1.23	0.722	1.11	0.859

U-OR depicts the unadjusted bivariate odds ratio

### Mental health outcomes

**Fig 1:**
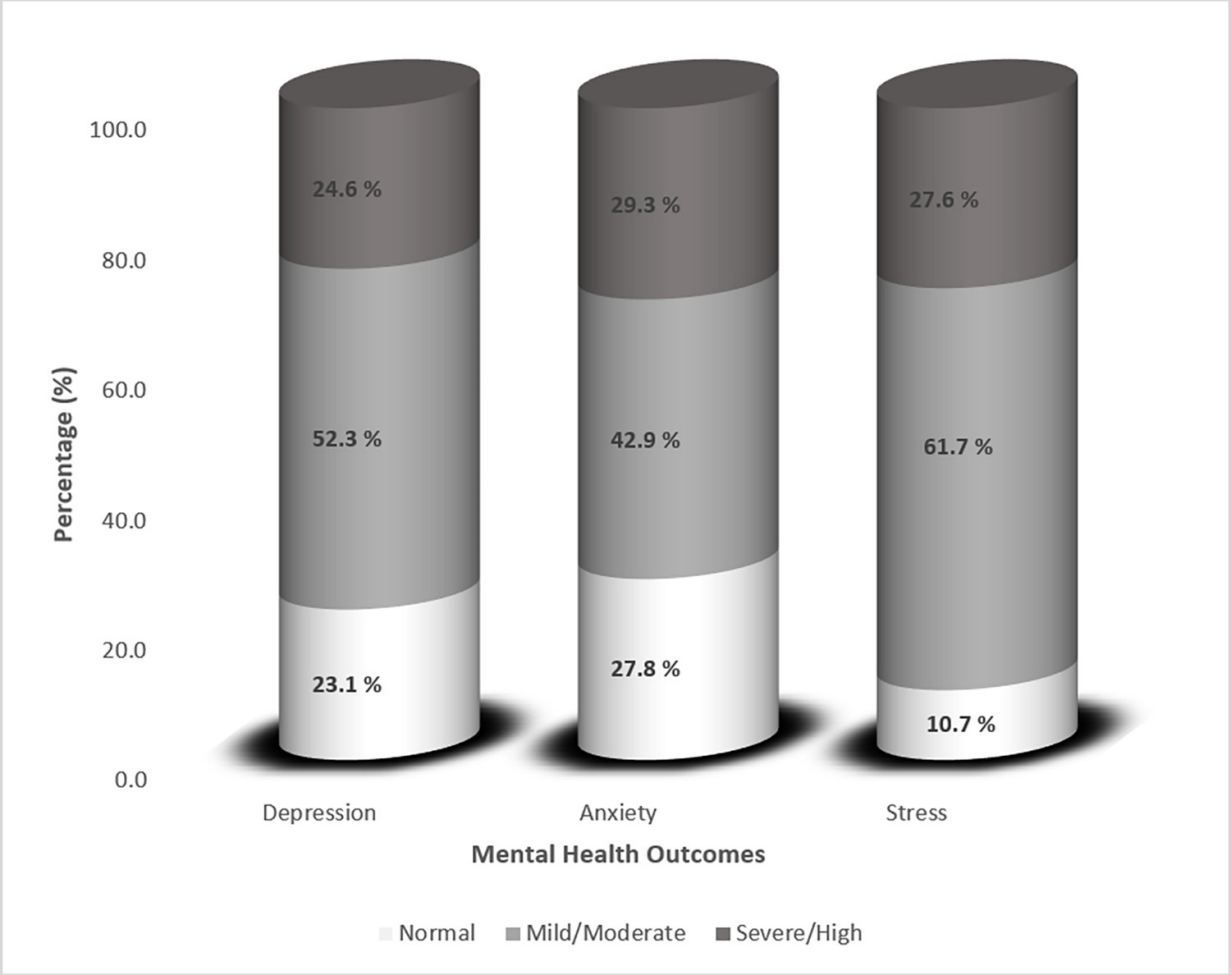
Depression, anxiety and stress levels among university students in Lebanon.

The mental health outcome scales were shown to have strong internal consistency (0.901 for depression, 0.944 for anxiety, and 0.846 for stress) in the study sample, as determined by Cronbach alpha. The mean (SD) score for depression was 10.18 (6.83), anxiety was 18.81 (14.42), and stress was 21.97 (7.30). Fig 1 depicts the study participants’ rates of depression, anxiety, and stress. Mild to moderate depression, anxiety, and stress were reported by the majority of participants (52.3%, 42.9%, and 61.7%, respectively), while severe depression, severe anxiety, and high stress were reported by 24.6%, 29.3%, and 27.6%, respectively. In total, students reported moderate to severe rates of depression, anxiety, and stress at a rate of 75.9%, 72.2%, and 89.3% respectively.

**Fig 2:**
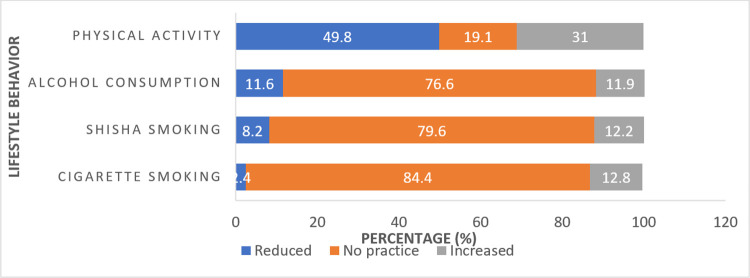
Lifestyle practices during the pandemic.

**Fig 3:**
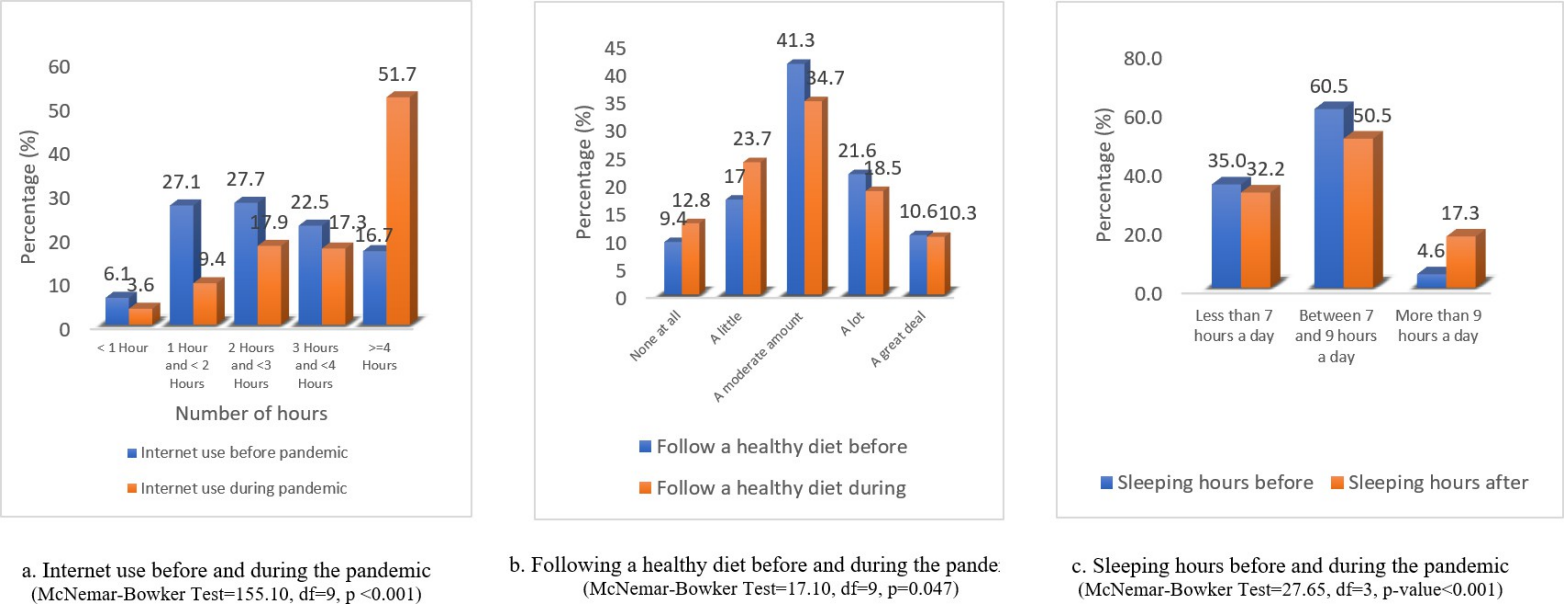
Changes in lifestyle practices.

Fig 2 highlights students’ lifestyle practices during the pandemic, whereas Fig 3 shows changes in lifestyle practices during the pandemic in comparison to before the pandemic. A significant difference in internet use, sleeping hours, and following a healthy diet was noted among university students during the pandemic (p-value = 0.001) as compared to before the pandemic. Tables 1 and 2 demonstrate the findings of the simple logistic regression analysis.

According to the results of multiple logistic regression analysis (Table 3) and after adjusting for sociodemographic and lifestyle factors, women have nearly three times the odds of anxiety and stress as men (AOR anxiety = 2.69, 95% CI: 1.20–5.75; p-value = 0.011; AOR stress = 2.93, 95% CI: 1.12–7.65; p-value = 0.028). University programs were linked to anxiety and stress. PhD students reported lower levels of anxiety than undergraduates (AOR anxiety/PhD program = 0.06, 95% CI: 0.01–0.71, p-value = 0.033).

**Table 3:** Adjusted multiple logistic regression analyses for the depression, anxiety and stress outcomes.

	Depression			Anxiety			Stress		
	A-OR	95% CI	P-value	A-OR	95% CI	P-value	A-OR	95% CI	P-value
**Age**	0.94	0.89–1.00	0.063	1.00	0.95–1.07	0.875	1.01	0.94–1.08	0.779
**Gender**									
Men	ref			ref			ref		
women	1.61	0.75–3.45	0.222	2.69	1.20–5.75	**0.011**	2.93	1.12–7.65	**0.028**
**Relationship status**									
Not in a relationship	1.08	0.85–1.37	0.528	1.12	0.88–1.43	0.339	1.17	0.90–1.53	0.249
In a relationship	ref			ref			ref		
**University program**									
Undergraduate degree	1.54	0.73–3.24	0.259	1.94	0.93–4.08	0.079	2.28	0.95–5.49	0.067
Certificate program	3.12	0.56–17.44	0.195	3.35	0.52–21.66	0.204	2.87	0.53–15.65	0.224
Graduate program (MA or MSc)	ref			ref			ref		
PhD Program	1.40	0.26–7.50	0.692	0.06	0.01–0.71	**0.026**	0.00	0.00-Inf	0.992
MD program	1.10	0.20–6.11	0.912	0.86	0.16–4.66	0.861	2.68	0.42–17.03	0.297
**GPA status during the pandemic**									
No change	1.14	0.54–2.43	0.729	1.48	0.68–3.22	0.322	0.88	0.36–2.11	0.768
Decreased	1.40	0.69–2.87	0.352	1.34	0.64–2.81	0.436	0.92	0.41–2.03	0.830
Increased	ref			ref			ref		
**Income (in USD)**									
≤450	0.85	0.40–1.82	0.675	2.00	0.93–4.30	0.078	0.94	0.38–2.32	0.885
>450	ref			ref			ref		
**Overall rated health**									
Poor	30.59	2.44–384.00	**0.008**	418.87	18.97–9251.40	**<0.001**	147067582.78	0.00-Inf	0.991
Fair	14.12	1.49–133.69	**0.021**	21.85	2.26–211.04	**0.008**	44692700.22	0.00-Inf	0.992
Good	4.42	0.49–40.27	0.187	4.47	0.48–41.43	0.187	16710160.79	0.00-Inf	0.992
Very good	3.17	0.33–30.14	0.315	7.67	0.81–72.92	0.076	16853535.43	0.00-Inf	0.992
Excellent	ref			ref			ref		
**Importance of religion in daily decisions**									
Not important	ref			ref			ref		
Important	0.46	0.20–1.10	0.080	1.06	0.44–2.52	0.904	0.38	0.15–0.97	**0.043**
**Conspiracy behind COVID virus/vaccine**									
Disapprove	0.65	0.26–1.60	0.346	1.42	0.56–3.63	0.461	0.53	0.18–1.52	0.235
Neither approve nor disapprove	1.00	0.41–2.44	0.993	1.68	0.66–4.28	0.275	0.83	0.31–2.23	0.706
Approve	ref			ref			ref		
**Adherence to COVID-19 preventive measures**									
No	1.22	0.52–2.89	0.643	1.34	0.55–3.24	0.516	0.35	0.12–1.01	0.051
Yes	ref			ref			ref		
**Infected with COVID-19**									
No	0.80	0.40–1.58	0.515	1.25	0.64–2.46	0.512	0.84	0.40–1.76	0.640
Yes	ref			ref			ref		
**Private Counseling**									
No	ref			ref			ref		
Yes	1.29	0.68–2.46	0.433	1.60	0.83–3.08	0.160	4.37	1.96–9.76	**<0.001**
**Follow healthy diet**									
No	1.27	0.65–2.49	0.492	0.91	0.45–1.82	0.784	1.82	0.86–3.85	0.117
Yes	ref			ref			ref		
**Cigarette smoking**									
No practice	ref			ref			ref		
Reduced	0.73	0.10–5.43	0.759	6.73	0.67–67.71	0.105	1.77	0.24–13.15	0.578
Increased	2.91	1.10–7.73	**0.032**	4.26	1.57–11.53	**0.004**	4.66	1.60–13.56	**0.005**
**Shisha smoking**									
No practice	ref			ref			ref		
Reduced	0.75	0.25–2.30	0.618	0.83	0.28–2.41	0.728	0.97	0.24–3.88	0.964
Increased	1.00	0.40–2.53	0.994	0.39	0.14–1.10	0.075	0.48	0.17–1.37	0.169
**Alcohol consumption**									
No practice	ref			ref			ref		
Reduced	0.52	0.16–1.70	0.278	0.22	0.06–0.85	**0.028**	0.24	0.06–1.02	0.054
Increased	0.43	0.14–1.27	0.127	0.96	0.33–2.82	0.943	0.29	0.08–1.05	0.060
**Physical activity**									
No practice	1.95	0.75–5.04	0.170	0.94	0.36–2.41	0.890	1.70	0.61–4.72	0.310
Reduced	1.85	0.90–3.80	0.095	1.29	0.62–2.65	0.496	2.42	1.05–5.59	**0.038**
Increased	ref			ref			ref		
**Sleeping hours**									
**<7**	3.19	1.54–6.61	**0.002**	1.65	0.80–3.40	0.174	0.87	0.38–1.99	0.744
7 to 9	ref			ref			ref		
>9	1.65	0.70–3.89	0.254	0.53	0.22–1.28	0.158	0.69	0.26–1.88	0.473
**Internet use (in hours)**									
<1	ref			ref			ref		
[1–2[	1.17	0.17–8.13	0.875	1.53	0.21–10.93	0.670	0.61	0.08–4.84	0.643
[2–3[	1.08	0.18–6.55	0.930	0.76	0.13–4.54	0.759	0.25	0.04–1.74	0.163
[3–4[	2.27	0.36–14.29	0.382	1.55	0.24–9.79	0.644	0.55	0.08–3.87	0.546
≥4	2.99	0.53–16.91	0.216	1.27	0.23–7.13	0.787	1.24	0.21–7.38	0.812

A-OR depicts the adjusted odds ratio of a model including each outcome with all of the sociodemographic factors and lifestyle practices.

Furthermore, depression and anxiety were linked to overall health rating, where the odds of depression are 30 and 418 times higher among those who rate their health as poor and fair, respectively, than among those who rate their health as excellent (AOR Depression/poor health = 30.59, 95% CI:2.44–384.00, p-value = 0.008; AOR Depression/Fair health = 14.12, 95% CI: 1.49–133.69, p-value = 0.021). When compared to those who rated their health as excellent, the odds of anxiety are 418 and 21 times higher for those who rated their health as poor and fair, respectively (AOR Anxiety/poor health = 418.87, 95% CI: 18.97–9251.40, p-value = <0.001; AOR Anxiety/Fair health = 21.85, 95% CI: 2.26–211.04, p-value = 0.008). Students who have said religion is important in their daily decisions also reported less stress than those who said religion is not important in their daily decisions (AOR Stress/Important = 0.38, 95%CI: 0.18–0.97, p-value = 0.043).

Additionally, stress was associated with private counseling, with those who sought private counseling having four-fold higher odds of stress than those who did not (AOR Stress = 4.37, 95% CI:1.96–9.76, p-value = <0.001). Cigarette smoking was associated with all mental health outcomes where individuals who increased their smoking intake during the pandemic, had nearly three time the odds of depression and four times the odds of anxiety and stress compared to those who did not smoke (AOR Depression = 2.91, 95%CI: 1.10–7.73, p-value = 0.032; AOR Anxiety = 4.26, 95% CI:1.57–11.53, p-value = 0.004; AOR Stress = 4.66, 95% CI: 1.60–13.56, p-value = 0.005). Students who reduced their alcohol consumption have lower odds of anxiety compared to those who do not consume alcohol (AOR anxiety = 0.22, 95%CI:0.06–0.85, p-value = 0.028). The odds of stress are two times higher among those who reduced their physical activity than in students who increased it (AOR Stress = 2.42, 95% CI: 1.05–5.59, p-value = 0.038). Students who slept fewer than seven hours per day had nearly three times the risk of depression than those who sleep seven to nine hours (AOR depression = 3.19, 95% CI: 1.54–6.61, p-value = 0.002). Finally, the Hosmer-Lemeshow p-values for the final models of depression, anxiety, and stress were 0.517, 0.893, 0.496, respectively, all of which are greater than 0.05, indicating adequate model fit.

All mental health outcomes had no significant association with the importance of religion in everyday decisions, adherence to COVID-19 measures, being previously infected with COVID-19, or internet use.

## Discussion

There is currently a scarcity of studies assessing the mental health of university students in Lebanon. This study aimed at understanding university students’ mental health, specifically depression, anxiety, and stress, during Lebanon’s extended COVID-19 pandemic, as well as the sociodemographic factors and lifestyle practices associated with it. University students are frequently regarded as the backbone of society, and their mental health during a pandemic may significantly impact their performance and life trajectories. Our results indicate that gender, university program, overall rated health, importance of religion in daily decisions, private counseling, smoking cigarettes, alcohol consumption, physical activity, and sleeping, are factors that influence these mental health outcomes.

In our study, moderate to severe rates of depression, anxiety, and stress were higher 2.2 times for depression, 2.3 times for anxiety, and 7 times for stress than previously reported rates among university students in Lebanon in April 2020 by Fawaz and Samaha in 2021. This might be explained by the increased economic downturn of the country where the devaluation of the money has almost doubled and it is known from pre-pandemic studies that depression, anxiety and stress have been associated with financial crisis [58, 59]. Another contributing factor might be the explosion of the Beirut port that destroyed a large area of the capital and impacted directly thousands of families with documented devastating mental health impact [60].

### Gender

Our findings point to a gender difference in symptoms of anxiety and stress, such as the odds of anxiety and stress were 2.7 and nearly 3 times higher among women compared to men. This is congruent with findings in other studies where being a female was found to be a risk factor for poor mental health among students [61–65].

### Self-rated health

Importantly, overall rated health stood out as the strongest predictor of depression and anxiety. Depression and anxiety were 30 times and 418 times, respectively, as high in the “poor” overall health group as those in the “excellent” group. While depression and anxiety were 14 times and 21 times as high in the “fair” overall health group as those in the “excellent” group. This is in line with other studies that uncovered that poor overall health was among the strongest predictors of these outcomes of depression and anxiety [66].

### Lifestyle practices

Nearly half of the students reported a decrease in physical activity, and this translated into a nearly 2.5 times increase in experienced stress among that group. Those results are consistent with other findings documenting a global shift in certain lifestyle practices. For example, the total physical activity in Italy fell dramatically during the first COVID-19 wave compared to before across all age categories, particularly in men [67]; however, changes in physical activities vary among countries. Studies from Belgium, France, and Switzerland, for instance, have found an overall rise in both physical activity [68, 69] and sedentary behavior [68]. This may be related to differences in cultural norms and/or governmental policies and investments that may support or hinder opportunities for physical activities during the pandemic; the European union has been known to take special care to implement policies promoting physical activity during the COVID-19 pandemic [70].

In terms of alcohol and smoking habits, this study shows an overall increase in cigarette and shisha smoking, as well as alcohol consumption during the COVID-19 pandemic. While shisha smoking had no effect on mental health outcomes, the increase in cigarette smoking worsened mental health outcomes for those who smoke by increasing the risk for depression, anxiety, and stress by nearly 3 times, 4 times, and 4.5 times, respectively, compared to non-smokers; this is in line with studies that found a positive association between smoking exposure and depression or anxiety [71]. Reduced alcohol consumption, on the other hand, was associated with significant reductions in anxiety. Existing research on alcohol consumption and smoking is mixed. Some studies have found no difference in alcohol intake during home confinement [72, 73] and a decrease in smoking [73, 74], while others found an increase in both alcohol consumption [75, 76] and smoking [75, 77]. However, it is worth noting that these studies reflect the situation at the early stages of the COVID-19 pandemic while our study was conducted two years afterwards.

Also, the odds of depression among students who slept less than 7 hours a day, were 3 times higher than in those who slept 7 to 9 hours a day; this is consistent with meta analysis studies that showed that sleep difficulty was significantly associated with depression [78, 79].

It is worthy to note that our study shows a significant decrease in following a healthy diet among university students during the pandemic as compared to before the pandemic; however, that was not translated with an impact on mental health. The literature is mixed as some studies have found minor changes in dietary behaviors [72, 80], while others depict an increase in unhealthy food consumption, overeating, and snacking in between meals [72, 73, 81].

### Religion and private counseling

Several studies found an association between the practice of religion and lower stress levels [82, 83], including during the COVID-19 pandemic [84]. In our study, the importance of religion in daily decisions was found to be significantly associated with a significant decrease in stress among study participants. Given the important role religion appears to play in Lebanese society [85], universities should take this into account while developing mental health programs. Furthermore, our findings imply that students who sought private counseling services were twice as likely as those who did not use private counseling services to experience worry and stress.

### Income

Finally, several studies have indicated that lower income contributes to poor mental health [64, 65] and that lower socioeconomic status has been linked to worsening in mental health [86, 87], including higher mortality and suicide rates, which are associated with economic downturns [88]. Our findings revealed no significant association between income and mental health outcomes, which is surprising given the well-established literature related to the social determinants of health. This could be due to the lack of variation in income among participants, as all local students were affected by the 90% local currency devaluation [89] and the fact that 85% of the respondents had an income of less than USD 1,000.

### Limitations of the study

This study has several limitations. First, given the cross-sectional nature of the study design, the results are subject to confounding biases such as the participants’ mental health status prior to the COVID-19 pandemic and other life stressors (e.g., experiences of violence). Second, there is the possibility of selection bias as participation was voluntary. Third, the study relied on a convenience sample limited to students from two universities. While this sampling technique does not necessarily assure that results are generalizable, it can be a useful tool for determining the likelihood of a potential relationship between the variables [90, 91]. Lastly, like any research conducted in an unstable environment with insecurity and instability, as well as constantly changing circumstances, predicting, and isolating the impact of these life factors is nearly impossible.

## Conclusion

We conducted a cross-sectional study among students from two universities in Lebanon. Our data reveal a national student mental health crisis, with exceptionally high rates of moderate to severe depression, anxiety, and stress. Our research also highlights the need of university administrators and mental health specialists paying close attention to the unique needs of female students, as well as those who have a poor or fair self-perceived health, as they are disproportionately affected by mental health issues. Specific programs addressing these categories are essential for their mental health, academic development, and economic contribution. Policies promoting physical activity may be crucial to develop when addressing mental health programming, and the role of religion in a student’s life may be a component to consider.

## References

[1] WHO. Coronavirus disease 2019 (COVID-19) Situation Report– 62. 2020.

[2] WHO. WHO Coronavirus (COVID-19) Dashboard: World Health Organization; 2022 [cited 2022 July 29]. Available from: https://covid19.who.int/.

[3] Mohapatra RK, Sarangi AK, Kandi V, Azam M, Tiwari R, Dhama K. Omicron (B. 1.1. 529 variant of SARS‐CoV‐2); an emerging threat: current global scenario. Journal of medical virology. 2021. 10.1002/jmv.27561 PMC9015454 34964506

[4] Zhai Y, Du X. Addressing collegiate mental health amid COVID-19 pandemic. Psychiatry research. 2020;288:113003. doi: 10.1016/j.psychres.2020.113003 32315885 PMC7162776

[5] Copeland WE, McGinnis E, Bai Y, Adams Z, Nardone H, Devadanam V, et al. Impact of COVID-19 Pandemic on College Student Mental Health and Wellness. Journal of the American Academy of Child & Adolescent Psychiatry. 2021;60(1):134–41. e2. 10.1016/j.jaac.2020.08.466 PMC8173277 33091568

[6] Ahmed O, Ahmed MZ, Alim SMAHM, Khan MAU, Jobe MC. COVID-19 outbreak in Bangladesh and associated psychological problems: An online survey. Death Studies. 2020:1–10. doi: 10.1080/07481187.2020.1818884 32915701

[7] El Othman R, Touma E, El Othman R, Haddad C, Hallit R, Obeid S, et al. COVID-19 pandemic and mental health in Lebanon: a cross-sectional study. Int J Psychiatry Clin Pract. 2021;25(2):152–63. doi: 10.1080/13651501.2021.1879159 33587678

[8] Brooks SK, Webster RK, Smith LE, Woodland L, Wessely S, Greenberg N, et al. The psychological impact of quarantine and how to reduce it: rapid review of the evidence. The lancet. 2020;395(10227):912–20. doi: 10.1016/S0140-6736(20)30460-8 32112714 PMC7158942

[9] Santomauro DF, Mantilla Herrera AM, Shadid J, Zheng P, Ashbaugh C, Pigott DM, et al. Global prevalence and burden of depressive and anxiety disorders in 204 countries and territories in 2020 due to the COVID-19 pandemic. The Lancet. 2021;398(10312):1700–12. doi: 10.1016/S0140-6736(21)02143-7 34634250 PMC8500697

[10] Taylor S, Landry CA, Paluszek MM, Fergus TA, McKay D, Asmundson GJG. COVID stress syndrome: Concept, structure, and correlates. Depress Anxiety. 2020;37(8):706–14. doi: 10.1002/da.23071 32627255 PMC7362150

[11] Laranjeira C, Dixe MA, Valentim O, Charepe Z, Querido A. Mental Health and Psychological Impact during COVID-19 Pandemic: An Online Survey of Portuguese Higher Education Students. International Journal of Environmental Research and Public Health. 2022;19(1):337. 10.3390/ijerph19010337 PMC8751187 35010604

[12] Ungar T. The health care payment game is rigged. National Post. 2015 April 28.

[13] Hawes MT, Szenczy AK, Klein DN, Hajcak G, Nelson BD. Increases in depression and anxiety symptoms in adolescents and young adults during the COVID-19 pandemic. Psychological Medicine. 2021:1–9. doi: 10.1017/S0033291720005358 33436120 PMC7844180

[14] Hou F, Bi F, Jiao R, Luo D, Song K. Gender differences of depression and anxiety among social media users during the COVID-19 outbreak in China: a cross-sectional study. BMC public health. 2020;20(1):1–11. 33148202 10.1186/s12889-020-09738-7 PMC7609822

[15] Cao W, Fang Z, Hou G, Han M, Xu X, Dong J, et al. The psychological impact of the COVID-19 epidemic on college students in China. Psychiatry research. 2020;287:112934. doi: 10.1016/j.psychres.2020.112934 32229390 PMC7102633

[16] Wang C, Wen W, Zhang H, Ni J, Jiang J, Cheng Y, et al. Anxiety, depression, and stress prevalence among college students during the COVID-19 pandemic: A systematic review and meta-analysis. Journal of American college health. 2021:1–8. doi: 10.1080/07448481.2021.1960849 34469261

[17] Krishnamoorthy Y, Nagarajan R, Saya GK, Menon V. Prevalence of psychological morbidities among general population, healthcare workers and COVID-19 patients amidst the COVID-19 pandemic: A systematic review and meta-analysis. Psychiatry research. 2020;293:113382. doi: 10.1016/j.psychres.2020.113382 32829073 PMC7417292

[18] Xiong J, Lipsitz O, Nasri F, Lui LM, Gill H, Phan L, et al. Impact of COVID-19 pandemic on mental health in the general population: A systematic review. Journal of affective disorders. 2020. 10.1016/j.jad.2020.08.001 PMC7413844 32799105

[19] Robinson E, Sutin AR, Daly M, Jones A. A systematic review and meta-analysis of longitudinal cohort studies comparing mental health before versus during the COVID-19 pandemic. medRxiv. 2021. 10.1016/j.jad.2021.09.098 PMC8578001 34600966

[20] Chinna K, Sundarasen S, Khoshaim HB, Kamaludin K, Nurunnabi M, Baloch GM, et al. Psychological impact of COVID-19 and lock down measures: An online cross-sectional multicounty study on Asian university students. PloS one. 2021;16(8):e0253059. doi: 10.1371/journal.pone.0253059 34343187 PMC8330936

[21] Wang X, Hegde S, Son C, Keller B, Smith A, Sasangohar F. Investigating mental health of US college students during the COVID-19 pandemic: cross-sectional survey study. Journal of medical Internet research. 2020;22(9):e22817. doi: 10.2196/22817 32897868 PMC7505693

[22] Aylie NS, Mekonen MA, Mekuria RM. The psychological impacts of COVID-19 pandemic among university students in Bench-Sheko Zone, South-west Ethiopia: a community-based cross-sectional study. Psychology Research and Behavior Management. 2020;13:813. doi: 10.2147/PRBM.S275593 33061696 PMC7533263

[23] Fawaz M, Samaha A, editors. E‐learning: Depression, anxiety, and stress symptomatology among Lebanese university students during COVID‐19 quarantine. Nursing Forum; 2021: Wiley Online Library. doi: 10.1111/nuf.12521 33125744

[24] Ghazawy ER, Ewis AA, Mahfouz EM, Khalil DM, Arafa A, Mohammed Z, et al. Psychological impacts of COVID-19 pandemic on the university students in Egypt. Health Promotion International. 2021;36(4):1116–25. doi: 10.1093/heapro/daaa147 33367587 PMC7799058

[25] Islam MA, Barna SD, Raihan H, Khan MNA, Hossain MT. Depression and anxiety among university students during the COVID-19 pandemic in Bangladesh: A web-based cross-sectional survey. PloS one. 2020;15(8):e0238162. doi: 10.1371/journal.pone.0238162 32845928 PMC7449469

[26] Lopes AR, Nihei OK. Depression, anxiety and stress symptoms in Brazilian university students during the COVID-19 pandemic: Predictors and association with life satisfaction, psychological well-being and coping strategies. PLoS one. 2021;16(10):e0258493. doi: 10.1371/journal.pone.0258493 34644347 PMC8513908

[27] Jaspal R, Assi M, Maatouk I. Potential impact of the COVID-19 pandemic on mental health outcomes in societies with economic and political instability: case of Lebanon. Mental Health Review Journal. 2020.

[28] Bou-Hamad I, Hoteit R, Harajli D. Health worries, life satisfaction, and social well-being concerns during the COVID-19 pandemic: Insights from Lebanon. Plos one. 2021;16(7):e0254989. doi: 10.1371/journal.pone.0254989 34324533 PMC8321151

[29] MOPH. Coronavirus COVID-19 Lebanon Cases 2022 [Available from: https://www.moph.gov.lb/en/Pages/127/27790/coronavirus-lebanon-cases-.

[30] Msheik El Khoury F, Talih F, Khatib MFE, Abi Younes N, Siddik M, Siddik-Sayyid S. Factors Associated with Mental Health Outcomes: Results from a Tertiary Referral Hospital in Lebanon during the COVID-19 Pandemic. Libyan J Med. 2021;16(1):1901438. doi: 10.1080/19932820.2021.1901438 33820499 PMC8032329

[31] Islam Z, Gangat SA, Mohanan P, Rahmat ZS, El Chbib D, Marfani WB, et al. Mental health impacts of Lebanon’s economic crisis on healthcare workers amidst COVID-19. Int J Health Plann Manage. 2021. doi: 10.1002/hpm.3324 34476840 PMC8652701

[32] Abed AE, Razzak RA, Hashim HT. Mental Health Effects of COVID-19 Within the Socioeconomic Crisis and After the Beirut Blast Among Health Care Workers and Medical Students in Lebanon. Prim Care Companion CNS Disord. 2021;23(4). doi: 10.4088/PCC.21m02977 34265874

[33] Fouad FM, Barkil-Oteo A, Diab JL. Mental Health in Lebanon’s Triple-Fold Crisis: The Case of Refugees and Vulnerable Groups in Times of COVID-19. Front Public Health. 2020;8:589264. doi: 10.3389/fpubh.2020.589264 33553090 PMC7855303

[34] El Chammay R, Roberts B. Using COVID-19 responses to help strengthen the mental health system in Lebanon. Psychol Trauma. 2020;12(S1):S281–s3. doi: 10.1037/tra0000732 32538651

[35] Younes S, Safwan J, Rahal M, Hammoudi D, Akiki Z, Akel M. Effect of COVID-19 on mental health among the young population in Lebanon. Encephale. 2021. 10.1016/j.encep.2021.06.007 PMC8426189 34583829

[36] Saadeh D, Sacre H, Hallit S, Farah R, Salameh P. Knowledge, attitudes, and practices toward the coronavirus disease 2019 (COVID‐19) among nurses in Lebanon. Perspectives in psychiatric care. 2021;57(3):1212–21. 33135217 10.1111/ppc.12676

[37] Domiati S, Itani M, Itani G. Knowledge, attitude, and practice of the Lebanese community toward COVID-19. Frontiers in Medicine. 2020;7:542. doi: 10.3389/fmed.2020.00542 33015096 PMC7461812

[38] Kroenke K, Spitzer RL, Williams JB. The PHQ‐9: validity of a brief depression severity measure. Journal of general internal medicine. 2001;16(9):606–13. doi: 10.1046/j.1525-1497.2001.016009606.x 11556941 PMC1495268

[39] Chin WY, Chan KT, Lam CL, Wong S, Fong DY, Lo YY, et al. Detection and management of depression in adult primary care patients in Hong Kong: a cross-sectional survey conducted by a primary care practice-based research network. BMC family practice. 2014;15(1):1–13. 24521526 10.1186/1471-2296-15-30 PMC3937039

[40] Manea L, Gilbody S, McMillan D. Optimal cut-off score for diagnosing depression with the Patient Health Questionnaire (PHQ-9): a meta-analysis. Cmaj. 2012;184(3):E191–E6. doi: 10.1503/cmaj.110829 22184363 PMC3281183

[41] Richardson LP, McCauley E, Grossman DC, McCarty CA, Richards J, Russo JE, et al. Evaluation of the Patient Health Questionnaire-9 Item for detecting major depression among adolescents. Pediatrics. 2010;126(6):1117–23. doi: 10.1542/peds.2010-0852 21041282 PMC3217785

[42] Zhang YL, Liang W, Chen ZM, Zhang HM, Zhang JH, Weng XQ, et al. Validity and reliability of P atient H ealth Q uestionnaire‐9 and P atient H ealth Q uestionnaire‐2 to screen for depression among college students in C hina. Asia‐Pacific Psychiatry. 2013;5(4):268–75. doi: 10.1111/appy.12103 24123859

[43] Sawaya H, Atoui M, Hamadeh A, Zeinoun P, Nahas Z. Adaptation and initial validation of the Patient Health Questionnaire–9 (PHQ-9) and the Generalized Anxiety Disorder–7 Questionnaire (GAD-7) in an Arabic speaking Lebanese psychiatric outpatient sample. Psychiatry research. 2016;239:245–52. doi: 10.1016/j.psychres.2016.03.030 27031595

[44] Beck AT, Epstein N, Brown G, Steer RA. An inventory for measuring clinical anxiety: psychometric properties. Journal of consulting and clinical psychology. 1988;56(6):893. doi: 10.1037//0022-006x.56.6.893 3204199

[45] Beck AT, Steer RA. Relationship between the Beck anxiety inventory and the Hamilton anxiety rating scale with anxious outpatients. Journal of Anxiety Disorders. 1991;5(3):213–23.

[46] Steer RA, Beck AT. Beck Anxiety Inventory. 1997.

[47] Beck AT, Epstein N, Brown G, Steer R. Beck anxiety inventory. Journal of consulting and clinical psychology. 1993. 10.1037//0022-006x.56.6.893 3204199

[48] Fydrich T, Dowdall D, Chambless DL. Reliability and validity of the Beck Anxiety Inventory. Journal of anxiety disorders. 1992;6(1):55–61.

[49] Al-Shatti TS. Psychometric properties of the Arabic Version of the Beck Anxiety Inventory in the State of Kuwait. Journal of Educational & Psychological Sciences. 2015;16(02).

[50] Cohen S, Kamarck T, Mermelstein R. A global measure of perceived stress. Journal of health and social behavior. 1983:385–96. 6668417

[51] Almeida LM, Costa-Santos C, Caldas JP, Dias S, Ayres-de-Campos D. The impact of migration on women’s mental health in the postpartum period. Revista de Saúde Pública. 2016;50:35. doi: 10.1590/S1518-8787.2016050005617 27355463 PMC4917335

[52] Makhubela M. Assessing psychological stress in South African university students: Measurement validity of the perceived stress scale (PSS-10) in diverse populations. Current Psychology. 2020:1–8.

[53] Andreou E, Alexopoulos EC, Lionis C, Varvogli L, Gnardellis C, Chrousos GP, et al. Perceived stress scale: reliability and validity study in Greece. International journal of environmental research and public health. 2011;8(8):3287–98. doi: 10.3390/ijerph8083287 21909307 PMC3166743

[54] Al-Dubai SAR, Alshagga MA, RAmpAL KG, Sulaiman NA. Factor structure and reliability of the Malay version of the perceived stress scale among Malaysian medical students. The Malaysian journal of medical sciences: MJMS. 2012;19(3):43. 23785249 PMC3684234

[55] El Rassoul AEA, Razzak RA, Hashim HT. Mental Health Effects of COVID-19 Within the Socioeconomic Crisis and After the Beirut Blast Among Health Care Workers and Medical Students in Lebanon. The Primary Care Companion for CNS Disorders. 2021;23(4):35348. 10.4088/PCC.21m02977 34265874

[56] Lu W, Bian Q, Wang W, Wu X, Wang Z, Zhao M. Chinese version of the Perceived Stress Scale-10: A psychometric study in Chinese university students. PloS one. 2017;12(12):e0189543. doi: 10.1371/journal.pone.0189543 29252989 PMC5734731

[57] Chaaya M, Osman H, Naassan G, Mahfoud Z. Validation of the Arabic version of the Cohen Perceived Stress Scale (PSS-10) among pregnant and postpartum women. BMC psychiatry. 2010;10(1):1–7. doi: 10.1186/1471-244X-10-111 21159169 PMC3016315

[58] Bartoll X, Palència L, Malmusi D, Suhrcke M, Borrell C. The evolution of mental health in Spain during the economic crisis. Eur J Public Health. 2014;24(3):415–8. doi: 10.1093/eurpub/ckt208 24367067

[59] Ruiz-Pérez I, Bermúdez-Tamayo C, Rodríguez-Barranco M. Socio-economic factors linked with mental health during the recession: a multilevel analysis. International journal for equity in health. 2017;16(1):1–8. 28264688 10.1186/s12939-017-0518-x PMC5339976

[60] Maalouf FT, Haidar R, Mansour F, Elbejjani M, Khoury JE, Khoury B, et al. Anxiety, depression and PTSD in children and adolescents following the Beirut port explosion. Journal of Affective Disorders. 2022;302:58–65. doi: 10.1016/j.jad.2022.01.086 35085669

[61] Schlichtiger J, Brunner S, Steffen J, Huber BC. Mental health impairment triggered by the COVID-19 pandemic in a sample population of German students. Journal of Investigative Medicine. 2020;68(8):1394–6. doi: 10.1136/jim-2020-001553 33087426

[62] Rogowska AM, Kuśnierz C, Bokszczanin A. Examining anxiety, life satisfaction, general health, stress and coping styles during COVID-19 pandemic in Polish sample of university students. Psychology Research and Behavior Management. 2020;13:797. doi: 10.2147/PRBM.S266511 33061695 PMC7532061

[63] Browning MH, Larson LR, Sharaievska I, Rigolon A, McAnirlin O, Mullenbach L, et al. Psychological impacts from COVID-19 among university students: Risk factors across seven states in the United States. PloS one. 2021;16(1):e0245327. doi: 10.1371/journal.pone.0245327 33411812 PMC7790395

[64] Margraf J, Brailovskaia J, Schneider S. Behavioral measures to fight COVID-19: An 8-country study of perceived usefulness, adherence and their predictors. Plos one. 2020;15(12):e0243523. doi: 10.1371/journal.pone.0243523 33284865 PMC7721173

[65] Kavčič T, Avsec A, Kocjan GZ. Psychological functioning of Slovene adults during the COVID-19 pandemic: does resilience matter? Psychiatric Quarterly. 2021;92(1):207–16. doi: 10.1007/s11126-020-09789-4 32556914 PMC7299145

[66] McCracken LM, Badinlou F, Buhrman M, Brocki KC. Psychological impact of COVID-19 in the Swedish population: Depression, anxiety, and insomnia and their associations to risk and vulnerability factors. Eur Psychiatry. 2020;63(1):e81. doi: 10.1192/j.eurpsy.2020.81 32843115 PMC7503043

[67] Maugeri G, Castrogiovanni P, Battaglia G, Pippi R, D’Agata V, Palma A, et al. The impact of physical activity on psychological health during Covid-19 pandemic in Italy. Heliyon. 2020;6(6):e04315. doi: 10.1016/j.heliyon.2020.e04315 32613133 PMC7311901

[68] Cheval B, Sivaramakrishnan H, Maltagliati S, Fessler L, Forestier C, Sarrazin P, et al. Relationships between changes in self-reported physical activity, sedentary behaviour and health during the coronavirus (COVID-19) pandemic in France and Switzerland. Journal of sports sciences. 2021;39(6):699–704. doi: 10.1080/02640414.2020.1841396 33118469

[69] Constandt B, Thibaut E, De Bosscher V, Scheerder J, Ricour M, Willem A. Exercising in times of lockdown: an analysis of the impact of COVID-19 on levels and patterns of exercise among adults in Belgium. International journal of environmental research and public health. 2020;17(11):4144. doi: 10.3390/ijerph17114144 32532013 PMC7312512

[70] World Health Organization. Physical activity promoting policies in the era of COVID-19: is Europe on the right track? Copenhagen, Denmark: World Health Organization; 2021 [updated October 4, 2021. Available from: https://www.euro.who.int/en/health-topics/disease-prevention/physical-activity/news/news/2021/10/physical-activity-promoting-policies-in-the-era-of-covid-19-is-europe-on-the-right-track.

[71] Fluharty M, Taylor AE, Grabski M, Munafò MR. The Association of Cigarette Smoking With Depression and Anxiety: A Systematic Review. Nicotine Tob Res. 2017;19(1):3–13. doi: 10.1093/ntr/ntw140 27199385 PMC5157710

[72] Ammar A, Brach M, Trabelsi K, Chtourou H, Boukhris O, Masmoudi L, et al. Effects of COVID-19 home confinement on eating behaviour and physical activity: results of the ECLB-COVID19 international online survey. Nutrients. 2020;12(6):1583. doi: 10.3390/nu12061583 32481594 PMC7352706

[73] Di Renzo L, Gualtieri P, Pivari F, Soldati L, Attinà A, Cinelli G, et al. Eating habits and lifestyle changes during COVID-19 lockdown: an Italian survey. Journal of translational medicine. 2020;18:1–15. 32513197 10.1186/s12967-020-02399-5 PMC7278251

[74] Elbay RY, Kurtulmuş A, Arpacıoğlu S, Karadere E. Depression, anxiety, stress levels of physicians and associated factors in Covid-19 pandemics. Psychiatry research. 2020;290:113130. doi: 10.1016/j.psychres.2020.113130 32497969 PMC7255248

[75] Stanton R, To QG, Khalesi S, Williams SL, Alley SJ, Thwaite TL, et al. Depression, anxiety and stress during COVID-19: associations with changes in physical activity, sleep, tobacco and alcohol use in Australian adults. International journal of environmental research and public health. 2020;17(11):4065. doi: 10.3390/ijerph17114065 32517294 PMC7312903

[76] Tran TD, Hammarberg K, Kirkman M, Nguyen HTM, Fisher J. Alcohol use and mental health status during the first months of COVID-19 pandemic in Australia. Journal of affective disorders. 2020;277:810–3. doi: 10.1016/j.jad.2020.09.012 33065821 PMC7476559

[77] Cancello R, Soranna D, Zambra G, Zambon A, Invitti C. Determinants of the lifestyle changes during COVID-19 pandemic in the residents of Northern Italy. International journal of environmental research and public health. 2020;17(17):6287. 32872336 10.3390/ijerph17176287 PMC7504331

[78] Li L, Wu C, Gan Y, Qu X, Lu Z. Insomnia and the risk of depression: a meta-analysis of prospective cohort studies. BMC Psychiatry. 2016;16(1):375. doi: 10.1186/s12888-016-1075-3 27816065 PMC5097837

[79] Baglioni C, Battagliese G, Feige B, Spiegelhalder K, Nissen C, Voderholzer U, et al. Insomnia as a predictor of depression: a meta-analytic evaluation of longitudinal epidemiological studies. J Affect Disord. 2011;135(1–3):10–9. doi: 10.1016/j.jad.2011.01.011 21300408

[80] Flanagan EW, Beyl RA, Fearnbach SN, Altazan AD, Martin CK, Redman LM. The impact of COVID‐19 stay‐at‐home orders on health behaviors in adults. Obesity. 2021;29(2):438–45. doi: 10.1002/oby.23066 33043562 PMC7675243

[81] Zajacova A, Jehn A, Stackhouse M, Denice P, Ramos H. Changes in health behaviours during early COVID-19 and socio-demographic disparities: a cross-sectional analysis. Canadian Journal of Public Health. 2020;111(6):953–62. doi: 10.17269/s41997-020-00434-y 33170494 PMC7654344

[82] Arévalo S, Prado G, Amaro H. Spirituality, sense of coherence, and coping responses in women receiving treatment for alcohol and drug addiction. Eval Program Plann. 2008;31(1):113–23. doi: 10.1016/j.evalprogplan.2007.05.009 17825910

[83] Peres MFP, Kamei HH, Tobo PR, Lucchetti G. Mechanisms Behind Religiosity and Spirituality’s Effect on Mental Health, Quality of Life and Well-Being. J Relig Health. 2018;57(5):1842–55. doi: 10.1007/s10943-017-0400-6 28444608

[84] Mahamid FA, Bdier D. The Association Between Positive Religious Coping, Perceived Stress, and Depressive Symptoms During the Spread of Coronavirus (COVID-19) Among a Sample of Adults in Palestine: Across Sectional Study. J Relig Health. 2021;60(1):34–49. doi: 10.1007/s10943-020-01121-5 33389439 PMC7778573

[85] Faour MA. Religion, demography, and politics in Lebanon. Middle Eastern Studies. 2007;43(6):909–21.

[86] Williams DR, Yu Y, Jackson JS, Anderson NB. Racial differences in physical and mental health: Socio-economic status, stress and discrimination. Journal of health psychology. 1997;2(3):335–51. doi: 10.1177/135910539700200305 22013026

[87] Murali V, Oyebode F. Poverty, social inequality and mental health. Advances in psychiatric treatment. 2004;10(3):216–24.

[88] Stuckler D, Basu S, McKee M. Budget crises, health, and social welfare programmes. Bmj. 2010;340. doi: 10.1136/bmj.c3311 20576709

[89] Reuters. Lebanon’s currency plummets again amid financial crisis and political deadlock: Reuters; 2022 [updated January 11, 2022. Available from: https://www.reuters.com/world/middle-east/lebanons-currency-plummets-again-amid-financial-crisis-political-deadlock-2022-01-11/.

[90] Bou-Hamad I. The impact of social media usage and lifestyle habits on academic achievement: Insights from a developing country context. Children and Youth Services Review. 2020;118:105425.

[91] Lim J, editor Considering the Impact of Self-regulation and Digital Literacy on Preserive Teachers’ Attitudes toward Web 2.0 Personal Learning Environment (PLEs). E-Learn: World Conference on E-Learning in Corporate, Government, Healthcare, and Higher Education; 2019: Association for the Advancement of Computing in Education (AACE).

