# Supplementary material for: A cross-sectional study of university students’ mental health and lifestyle practices amidst the COVID-19 pandemic
Source: PLoS One. 2024 Apr 16;19(4):e0302265. doi: 10.1371/journal.pone.0302265 (PMC11020786; doi:10.1371/journal.pone.0302265)
Supplement: Attachment — Submitted filename: Response to Reviewers.docx [file pone.0302265.s002.docx]

22-March-2024

PLOS ONE

Re Manuscript ID: PONE-D-23-37343R1 entitled “A Cross-sectional Study of University Students' Mental Health and Lifestyle practices Amidst the COVID-19 Pandemic.”

We would like to thank the editor and reviewers for the opportunity to review our manuscript entitled: “A Cross-sectional Study of University Students' Mental Health and Lifestyle practices Amidst the COVID-19 Pandemic.”

We deeply appreciate all the helpful feedback and suggestions that helped us enhance the article's content. We responded to all of the journal’s and reviewers’ comments and made significant improvements.

**Our responses (in bold black and blue, with blue indicating newly included or modified statements in the paper) are described below in a point-to-point manner.**

**Journal requirements:**

When submitting your revision, we need you to address these additional requirements.

1. Please review your reference list to ensure that it is complete and correct. If you have cited papers that have been retracted, please include the rationale for doing so in the manuscript text, or remove these references and replace them with relevant current references. Any changes to the reference list should be mentioned in the rebuttal letter that accompanies your revised manuscript. If you need to cite a retracted article, indicate the article’s retracted status in the References list and also include a citation and full reference for the retraction notice.

**Authors’ response:**

**We have carefully reviewed our reference list and made the necessary adjustments as per your instructions. Specifically, we have replaced the reference to the article titled "Reliability and validity of the Patient Health Questionnaire-9 in Chinese adolescents" by Hu et al. with another relevant reference:**

**Original Reference:** Hu X, Zhang Y, Liang W, Zhang H, Yang S. Reliability and validity of the patient health questionnaire-9 in Chinese adolescents. Sichuan Ment Health. 2014;27(4):357-60.

**Replacement Reference: Reference #42:** Zhang YL, Liang W, Chen ZM, Zhang HM, Zhang JH, Weng XQ, et al. Validity and reliability of Patient Health Questionnaire-9 and Patient Health Questionnaire-2 to screen for depression among college students in China. Asia-Pacific Psychiatry. 2013;5(4):268-75.

**Reviewer #1:**

Reviewer #1: It is suggested that authors delete the following sentence "Scalable solutions, such as online mindfulness (Ahmad et al., 2018; El Morr et al., 2017; ElMorr et al., 2020), are also important to investigate in order to alleviate the mental health crisis among university students in Lebanon" from the Conclusion, as the content is not properly addressed in the manuscript. Furthermore, references have no place in Conclusions.

**Authors’ response:**

**Thank you. Please note that we have deleted the following sentence from the conclusion.**

“Scalable solutions, such as online mindfulness [92-94], are also important to investigate in order to alleviate the mental health crisis among university students in Lebanon.”

**Reviewer #2: (No Response)**

**Authors’ response:**

**Thank you for taking the time to review our manuscript.**
